# Comprehensive Transcriptome–Metabolome Analysis and Evaluation of the *Dark_Pur* Gene from *Brassica juncea* that Controls the Differential Regulation of Anthocyanins in *Brassica rapa*

**DOI:** 10.3390/genes13020283

**Published:** 2022-01-31

**Authors:** Yujia Liu, Guoliang Li, Shujiang Zhang, Shifan Zhang, Hui Zhang, Rifei Sun, Fei Li

**Affiliations:** Institute of Vegetables and Flowers, Chinese Academy of Agricultural Sciences, Beijing 100081, China; carrotlyj@163.com (Y.L.); liguoliang@caas.cn (G.L.); zhangshujiang@caas.cn (S.Z.); zhangshifan@caas.cn (S.Z.); zhanghui05@caas.cn (H.Z.); sunrifei@caas.cn (R.S.)

**Keywords:** anthocyanin, purple chinese cabbage, purple mustard, LC-MS/MS, RNA-seq, transformation

## Abstract

Chinese cabbage (*Brassica rapa*) is a major vegetable crop in China. The accumulation of anthocyanins improves the quality and flavor of *Brassica* crops and is beneficial for human health. There has been great research interest in breeding purple Chinese cabbage, for which it is necessary to study the key genes and mechanisms of anthocyanin accumulation. Through distant hybridization between purple mustard (*Brassica juncea*) and green Chinese cabbage (*B. rapa*), purple Chinese cabbage plants were obtained. Furthermore, the *Dark_Pur* gene was cloned in the purple Chinese cabbage plants, which came from purple mustard and may be responsible for the purple phenotype in purple Chinese cabbage plants. Through particle bombardment of isolated microspores from Chinese cabbage to transform the *Dark_Pur* gene, the transformed purple Chinese cabbage plant was obtained, thus verifying the function of the *Dark_Pur* gene. To further study the *Dark_Pur* gene regulatory mechanism of anthocyanin accumulation in Chinese cabbage, the purple/green Chinese cabbage lines and purple/green mustard lines were subjected to transcriptome–metabolome analysis. Three stages (cotyledon, seedling, and large-leaf stages) of the purple/green Chinese cabbage lines and purple/green mustard lines were selected for analysis. The results indicated that the expression level of the transcription factor genes *BraA09g028560.3C, BraA03g019460.3C,* and *BraA07g035710.3C* may be induced by the *Dark_Pur* gene and they play an important role in purple Chinese cabbage, and *BjuB010898 and BjuO006089* may be responsible for anthocyanin accumulation in mustard. Studying the structural genes of the purple Chinese cabbage showed that *PAL*, *C4H*, *4CL*, *CHS*, *CHI*, *F3H*, *F3’H*, *FLS*, *DFR*, *ANS*, and *UGT* were up-regulated in three growth periods. There were 22 and 10 differentially expressed metabolites (DEMs) in seedling and large-leaf stages between purple/green Chinese cabbage, respectively, and 12 and 14 differentially expressed metabolites (DEMs) in seedling and large-leaf stages between purple/green mustard, respectively, which may indicate that the *Dark_Pur* gene from purple mustard greatly regulates anthocyanin accumulation in purple Chinese cabbage. This study provides a foundation for further elucidating anthocyanin regulation.

## 1. Introduction

Anthocyanins are flavonoids and important water-soluble pigments that make up the color of plants. There are six common anthocyanins, namely, cyanidin, pelargonidin, peonidin, malvidin, petunidin, and delphidin [1]. Studies have shown that anthocyanins have health care functions relating to their strong antioxidant capacity, which can help prevent cancer, cardiovascular, and cerebrovascular diseases [2,3]. In recent years, the genetic traits of purple coloring, anthocyanin composition, gene mapping, related gene expression, anthocyanin expression patterns and associated genes have been studied [4,5].

The mechanisms of anthocyanin biosynthesis and regulation in model species such as *Arabidopsis*, *Zea mays*, and *Petunia hybrida* have been well studied [6,7]. Research on the genetic and molecular mechanisms of anthocyanin biosynthesis shows that this process involves three main steps. First, PAL (phenylalanine ammonia lyase), C4H (cinnamate 4-hydroxylase), and 4CL (4-coumarate: CoA ligase) are involved in the phenylpropanoid metabolic pathway. Second, early biosynthetic genes (EBGs), including CHS (chalcone synthase), CHI (chalcone isomerase), F3H (flavanone 3-hydroxylase), F3′H (flavanone 3’-hydroxylase), and FLS (flavonol synthase) provide precursor substrates for the synthesis of flavonols and anthocyanins. The late biosynthetic genes (LBGs), including DFR (dihydroflavonol 4-reductase), ANS (anthocyanin synthase), and UGT (UDP-glucosyltransferase), are essential for producing anthocyanins and certain flavonoids [8,9,10,11,12,13]. Some studies show that LBGs are regulated by the MBW complex, which consists of MYB, bHLH, and WD40 transcription factors, and EBGs are usually activated by the R2R3-MYB transcription factor [14,15]. In *Arabidopsis*, the *AtPAP1, AtMYB113*, *AtMYB114, AtTT8,* and *AtTTG1* genes may participate in MBW formation [6]. Overexpression of *AtPAP1*, *AtMYB113*, and *AtMYB114* can increase the production of anthocyanin in a TTG1- and bHLH-cooperative manner, and the *TT2, TT8,* and *TTG1* genes can activate proanthocyanin biosynthesis in *Arabidopsis* seeds [16,17]. Moreover, negative regulators also play an important role in the formation of anthocyanins. *MYBL2* and *CPC*, and *LBD37, LBD38,* and *LBD39* are negative regulators of anthocyanin biosynthesis in *Arabidopsis* [18,19]. With the ability to inhibit the biosynthetic genes or directly inhibit the activity of the MBW complex, *AtMYBL2* and *AtCPC* inhibit the accumulation of anthocyanins [20]. The key regulatory genes *AtPAP1* and *AtPAP2* were suppressed by the negative regulators *AtLBD37, AtLBD38,* and *AtLBD39* in anthocyanin biosynthesis in *Arabidopsis* [19,21]. Previous findings suggest that both activators and repressors are important in the anthocyanin biosynthesis pathway.

At present, the research on *Brassica* anthocyanins has mainly focused on the location of related genes and the cloning and functional verification of certain genes in the synthesis pathway. The gene loci of anthocyanins in *Brassica* species have been mapped, and the loci present in various species have been explored. The gene *BoMYB2* is considered to be the major contributor to the coloring of purple cauliflower [22]. A yeast two-hybrid assay verified that the protein encoded by the *BoMYB2* gene in cauliflower could interact with bHLH and WD40 to form a complex and could up-regulate the expression of *AtTT8, AtEGL3, F3’H, DFR, ANS,* and other genes. The genes *BrbHLH49*, *BrEGL3.2,* and *BrMYBL2.1* are considered to be crucial for coloration in zicaitai [23,24,25,26]. In red leaf cabbage, *BoMYB2* or *BoMYBL2.1* seems to be important [27,28]. *BrTT8* is considered to be a candidate gene in purple bok choy [29]. In purple *B. rapa*, the gene *BrMYB2* and the R2R3-MYB transcript *c3563g1i2* from the B genome of *Brassica* are considered to be crucial [30,31]. *BjP11* and *BjPur* are the candidate genes in *B. juncea* [32,33]. Research shows that in the anthocyanin biosynthesis pathway in *Brassica*, the mechanism of ABGs (anthocyanin biosynthesis genes) activated by the MBW ternary complex is similar to that of the mentioned plants [16]. Purple Chinese cabbage is usually bred from other purple varieties, and so the genes are located on different chromosomes. A previous study considered that the purple gene was located on A02 of purple *B. rapa*, while another study considered that the purple gene was located on A07 [34,35,36]. There is a lack of research of anthocyanin regulation in purple Chinese cabbage, and thus, the anthocyanin accumulation mechanisms of purple *B. rapa* need to be explored.

In our previous study, purple Chinese cabbage was obtained from the distant hybridization between purple *B. juncea* (AABB) and green Chinese cabbage (AA) through embryo rescue [30]. Through genetic and transcriptome analysis, the candidate transcript *c3563g1i2* was gained, which might be responsible for the purple phenotype [30]. In this study, four lines, namely, purple *B. rapa* ‘2217-Pur’ and green *B. rapa* ‘2217-Gre’ (‘2217-Pur’ and ‘2217-Gre’ separated from the 2217 highly inbred line) and purple *B. juncea* ‘B90830’ and green *B. juncea* ‘2116’ were used as experimental materials. Based on their predecessors, the insert sequence of *Dark_Pur* and its promoter sequence were cloned in the four materials above, and the function of the gene was verified through a transformation system. To further study whether the *Dark_Pur* induced the purple coloration mechanism of *B. rapa*, transcriptome–metabolome joint analysis was conducted. The goal of this study is to explore the mechanism of anthocyanin accumulation in Chinese cabbage and provide a basis for purple Chinese cabbage breeding in the future. The regulatory mechanism of purple coloration during distant hybridization between *Brassica* species is also analyzed.

## 2. Materials and Methods

### 2.1. Plants Materials

Four materials were chosen, and their names are ‘2217-Pur’, ‘2217-Gre’, ‘B90830’, and ‘2116’. The mustard ‘B90830’ was used as the male parent and hybridized with another green Chinese cabbage. Picking the offspring and through eight continuous rounds of selfing, highly inbred line 2217 was obtained. The *B. rapa* ‘2217-Pur’ and ‘2217-Gre’ were separated from the inbred line 2217. The ‘2116’ is green mustard, which has a similar appearance to purple mustard ‘B90830’. The ‘2217-Pur’ is dark purple; the color of the underside of the leaf is lighter than that of the upper leaf. By contrast, ‘2217-Gre’ is completely green, both on the up- and undersides of the leaves. The *B. juncea* ‘B90830’ is a purple material with a spotty dark purple pattern on the leaf surface, and the veins on the back of the leaf are also purple. The green *B. juncea* ‘2116’ group is also completely green. The cotyledon stage (choose the cotyledons when there were four true leaves), seedling stage (about six true leaves), and large-leaf stage (about 10 true leaves) were chosen as the experimental periods. The three stages of ‘2217-Gre’ were named GC-C, GC-I, and GC-II, and the three stages of ‘2217-Pur’ were named PC-C, PC-I, and PC-II. The three stages of *B. juncea* ‘B90830’ were named PM-C, PM-I, and PM-II, and the three stages of ‘2116’ were named GM-C, GM-I, and GM-II.

### 2.2. DNA Extraction, RNA Extraction, and Reverse Transcription

The CTAB method was used to extract the plant DNA from leaf samples that were frozen at −80 °C [30]. Using an RNA extraction reagent test kit (Gene better R218-50, Beijing, China), RNA could be easily obtained at room temperature. After testing the RNA quality, a reverse transcription reagent test kit (Trans AE311, Beijing, China) was used to obtain cDNA.

### 2.3. Plant Genetic Transformation

The cDNA of the candidate gene was cloned, using gene-specific primers (Table S1) and transferred into the pCAMBIA2300 vector. The content of the insert section of cDNA is 29.16 ng and its ratio to the vector is 2:1. CaMV35S promoter, eGFP and Kanr labels are present in the pCAMBIA2300. Using *B. rapa* microspores as receptors, gold particle-plasmid DNA bombardment microprojectiles were prepared for genetic transformation, using the particle bombardment method. NLN-13 medium and B5 medium were used for culturing. The regenerated plants were subjected to phenotypic identification and PCR verification to obtain transgenic plants. The 10 μL system was used for the PCR process, and the procession of the PCR process was as follows. The first step is 95 °Cfor 3 min; the second step is 95 °C for 15 s, 56 °Cfor 15 s, and 72 °C for 15 s; and the last step is 72 °C for 5 min. The second step included 35 cycles. The GeneAmp^®^ PCR System 9700 (Applied Biosystems, Carlsbad, CA, USA) was used for the PCR procession.

### 2.4. Sequence Analysis of the Candidate Gene and Its Promoter

The snapgene and Exon-Intron Graphic Maker online (http://www.wormweb.org/exonintron, accessed on 29 September 2021) were used to analyze the gene structure of exons and introns. SMART (http://smart.embl-heidelberg.de/, accessed on 29 September 2021), Expasy (http://web.expasy.org/protparam/, accessed on 29 September 2021) and phyre2 (http://www.sbg.bio.ic.ac.uk/phyre2/html/page.cgi?id=index, accessed on 29 September 2021) were used for amino acid domain, hydrophobicity, and protein tertiary structure prediction analysis. DNA sequence alignment analysis was also conducted using SnapGene software (https://www.snapgene.cn, accessed on 29 September 2021).

### 2.5. Sample Collection and RNA Sequencing

Samples from each period were stored at −80 °C for RNA extraction. Leaf samples were used for total RNA extraction, and an RNA extraction kit (TIANGEN, Beijing, China) was used following the manufacturer’s protocol. The NanoDrop 2000 spectrophotometer (Thermo Fisher Scientific, Wilmington, DE, USA) was used for assessing the purity, and the RNA Nano 6000 Assay Kit of the Agilent Bioanalyzer 2100 system (Agilent Technologies, Santa Clara, CA, USA) was used for evaluating the integrity of the RNA. According to the manufacturer’s instructions, a total amount of 1 μg RNA per sample was used for the RNA sample preparations, and 36 sequencing (4 accessions × 3 periods × 3 replicates) libraries were generated using NEBNext UltraTM RNA Library Prep Kit for Illumina (NEB, Ipswich, MA, USA). The library concentration of the study requires ≥ 5 nM, volume > 100 uL. The experiment was operated by Biomarker Technologies Corporation, Beijing, China.

### 2.6. Transcriptome Data Preparation

The reference genome sequence (v1.5) of the *B. juncea* (genome size, 954,861,368 bp) and the sequence (v3.0) of *B. rapa* (genome size, 356,687,492 bp) were downloaded from the Brassica Database (http://39.100.233.196/#/Download/, accessed on 29 September 2021) [37,38]. There are 9746 scaffolds with a scaffold N50 of 38.8 Mb and 18,588 contigs, with a contig N50 of 0.18 Mb in sequence (v1.5) of *B. juncea*. There are 1301 scaffolds with a scaffold N50 of 4.44 Mb and 1476 contigs, with a contig N50 of 1.45 Mb in sequence (v3.0) of *B. rapa*. The Q20, Q30, GC-content, and sequence duplication level from clean data were calculated. Hisat2 tools and the FPKM were used to map the reference genome and estimate the gene expression levels (http://ccb.jhu.edu/software/hisat2/index.shtml, accessed on 29 September 2021) [39]. All the downstream analyses were based on high quality clean data.

### 2.7. Differential Gene Expression Analysis

The DEG analysis of two groups of *B. rapa* and two groups of *B. juncea* was performed using DESeq2 (http://www.bioconductor.org/packages/release/bioc/html/DESeq.html, accessed on 29 September 2021) [40]. The Benjamini and Hochberg’s were used to adjust the resulting *P*-values and control the FDR. In the process of detecting differentially expressed genes, FDR < 0.01 and fold-change ≥ 2 were used as the screening criteria. Heatmaps were produced by TBtools (https://www.tbtools.com, accessed on 29 September 2021) [41].

### 2.8. Gene Functional Annotation and Enrichment Analysis

KOG/COG (clusters of orthologous groups of proteins), KO (KEGG ortholog database), Swiss-Prot (a manually annotated and reviewed protein sequence database) were used to annotate the gene function. GO enrichment analysis of the DEGs was implemented by the GOseq R package based on the Wallenius non-central hyper-geometric distribution (http://www.bioconductor.org/packages/release/bioc/html/goseq.html, accessed on 29 September 2021) [42]. KEGG was used to elucidate the advanced functions and utilities of the biological system (http://www.genome.jp/kegg/, accessed on 29 September 2021) [43]. KOBAS software was used to test the DEGs statistical enrichment in KEGG pathways (http://kobas.cbi.pku.edu.cn/genelist/, accessed on 29 September 2021) [44].

### 2.9. Quantitative Real Time PCR

RNA samples were reverse transcribed to cDNA, using HiScript III All-in one RT SuperMix Perfect (Vazyme, Nanjing, China) for qPCR. Relative expression level of the DEGs were detected through quantitative real-time PCR using Taq Pro Universal SYBR qPCR Master Mix (Vazyme, Nanjing, China) and CFX-96 Real-time System (BIORAD, Hercules, CA, USA). After the PCR reactions, the curves analysis was carried out. Using the the 2^−∆∆CT^ method, the relative expression levels were calculated [45]. The primer sequences used in the qPCR analysis are shown in Table S2.

### 2.10. Sample Preparation and Metabolite Extraction

Samples at the same stage as the samples collected for RNA-seq were stored at −80 °C for metabolite extraction. The two *B. rapa* lines ‘2217-Pur’ and ‘2217-Gre’ were divided into two periods, namely, the seedling stage and large-leaf stage, for metabolite analysis. The two *B. juncea* lines ‘B90830’ and ‘2116’ were also divided into two periods, namely, the seedling stage and large-leaf stage, for metabolite analysis.

According to the manufacturer’s protocol, 24 metabolite samples were thawed at 4 °C on ice. Then 300 μL of methanol was added to extract 100 μL of the sample. Twenty microliters of internal standard was added, followed by vortexing for 30 s. Then, the samples were sonicated for 10 min (incubated in ice water) and incubated for 1 h at −20 °C to precipitate the proteins. After being centrifuged at 13,000 rpm for 15 min at 4 °C, the supernatant was used for the LC-MS/MS analysis. The extract analysis was performed by BioMarker (Beijing, China).

### 2.11. LC-MS/MS Analysis and Metabolite Profiling Analysis

A UHPLC system (1290 Agilent Technologies, Santa Clara, CA, USA) with a UPLC BEH Amide column (1.7 μm 2.1 * 100 mm, Waters) coupled to TripleTOF 5600 (Q-TOF, AB Sciex, Framingham, MA, USA) was used to analyze the LC-MS/MS. The mobile phase consisted of 25 mM NH4OAc and 25 mM NH4OH in water (pH = 9.75) (A), and acetonitrile (B) was carried with an elution gradient as follows: 0 min, 95% B; 7 min, 65% B; 9 min, 40% B; 9.1 min, 95% B; 12 min, 95% B. This was delivered at 0.5 mL min^−1^. The ProteoWizard and R package XCMS (https://bioconductor.org/packages/release/bioc/html/xcms.html, accessed on 29 September 2021) were used to deal the MS raw data files. The XCMS data were peaked using R package CAMERA, and the metabolites were identified using an in-house MS2 database. QC analysis was performed to confirm the reliability of the data. Metabolites with a fold-change ≥ 1, *p*-value < 0.05, and variable importance in the projection (VIP) > 1 were considered DEMs. Metabolite analysis was performed using BMKCloud (http://www.biocloud.net, accessed on 29 September 2021).

## 3. Results

### 3.1. Purple Phenotype Analysis in B. rapa and B. juncea

**Figure 1:**
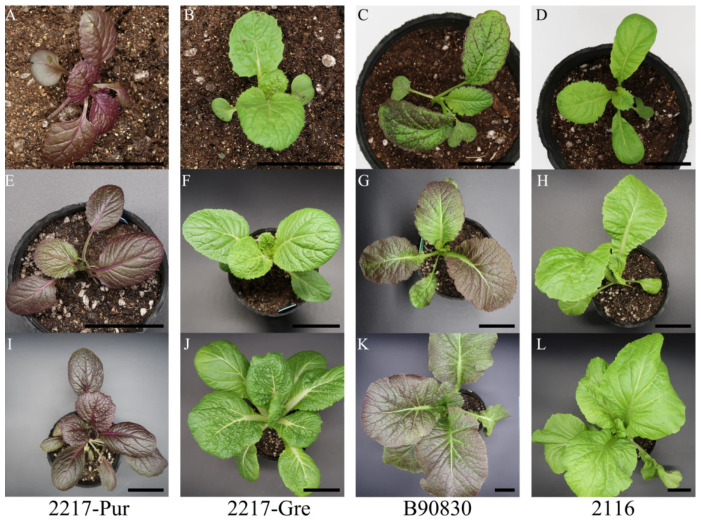
Different periods of the different materials. (**A**–**D**): cotyledon stage of ‘2217-Pur’, ‘2217-Gre’, ‘B90830’ and ‘2116’; (**E**–**H**): seedling stage of ‘2217-Pur’, ‘2217-Gre’, ‘B90830’ and ‘2116’; (**I**–**L**): large-leaf stage of ‘2217-Pur’, ‘2217-Gre’, ‘B90830’ and ‘2116’. The scale bar is 3 cm (**A**–**D**) and 5 cm (**E**–**L**).

There were differences between the purple and green groups of *B. rapa* and *B. juncea* in three stages: the cotyledon stage, seedling stage, and large-leaf stage. As indicated in Figure 1, the purple *B. rapa* ‘2217-Pur’ began to turn purple once its cotyledon emerged, while the green *B. rapa* remained green. The up surface of the Chinese cabbage was completely purple, and the veins on the down surface were also purple. The color of ‘2217-Pur’ became darker as time progressed. As observed with the purple *B. rapa*, the purple *B. juncea* ‘B90830’ began to turn purple once its cotyledon emerged, while the green mustard ‘2116’ remained green. However, ‘B90830’ showed a spotty purple phenotype compared with ‘2217-Pur’.

**Figure 2:**
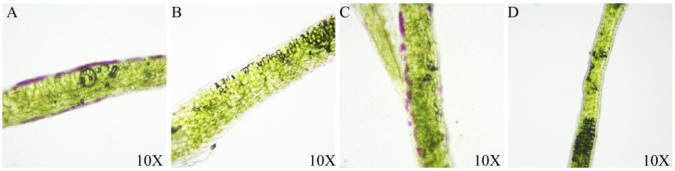
Cross sections of the different materials. (**A**–**D**): 2217-Pur, 2217-Gre, B90830, 2116.

To test anthocyanin distribution in the leaves of *B. rapa* and *B. juncea*, cross sections of blades of different materials were made (Figure 2). The purple materials both exhibited anthocyanin accumulation on the upper and lower surfaces, and the anthocyanin content of the upper epidermis was higher than that of the lower epidermis in both *B. rapa* and *B. juncea*. By contrast, the green materials did not exhibit any anthocyanin accumulation on either side of the leaves. The different shades of purple were related to the accumulation of anthocyanins on the surface of the leaves.

### 3.2. Cloning and Characteristic Analysis of the Dark_Pur Gene

**Figure 3:**
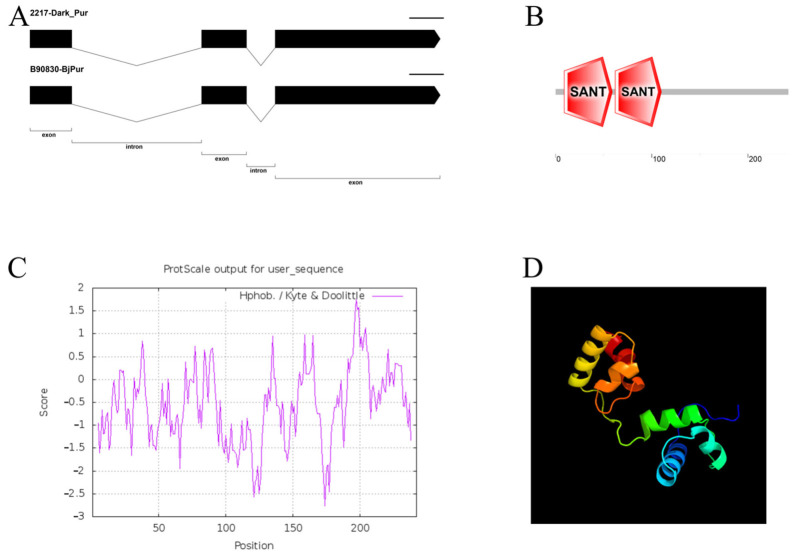
Characteristic of analysis of *Dark_Pur* gene sequence and protein sequence. (**A**) Comparison of exons and introns of *Dark_Pur.* (**B**) Protein domain analysis. (**C**) Hydrophobic structure of protein. (**D**) Protein tertiary structure prediction.

In our previous research, the candidate gene *c3563g1i2* was screened through initial location and transcriptome analysis [30,34]. In this study, we performed a cloned gene structure analysis of this gene. A special pair of *B. rapa* inbred lines (‘2217-Pur’, and ‘2217-Gre’) were chosen as the experimental materials. Another *B. juncea* line containing both purple and green materials was also chosen. The CTAB method was used to extract the DNA from the tissue from the two groups, and an RNA extraction reagent test kit was used to extract the plant RNA [30]. The gDNA and cDNA samples were subjected to PCR. The primers used are listed in Table S1. Compared with purple *B. juncea*, *B. rapa* had a deeper purple color, and thus the gene was named *Dark_Pur*. The gDNA and cDNA sequences were compared, and the structure of *Dark_Pur* in *B. rapa* and *B. juncea* was determined. Using the gDNA as a template, the PCR detection of the experimental materials ‘2217-Pur’, ‘2217-Gre’, ‘B90830’, and ‘2116’ revealed that the purple materials (‘2217-Pur’, ‘B90830’) and green mustard (‘2116’) had bands of 1188 bp in length, while the green Chinese cabbage did not have any bands. The sequences between the different materials were also compared. Sequence analysis showed that *Dark_Pur* of ‘2217-Pur’ and *Bjpur* of ‘B90830’ had the same sequences, and the sequence was the same as in the reference genome of *B. juncea* (Figure 3A). The sequences had three exons and two introns, while the green Chinese cabbage materials did not have any sequences. Analysis of the DNA domains (Figure 3B) indicated that there were two specific SANT (the important functional areas) hit regions. SANT domains are present in the nuclear receptor co-repressors and subunits of many chromatin-remodeling complexes. It shares strong structural similarity with the DNA-binding domain of Myb-related proteins. Despite the overall similarity, there are differences, suggesting that the SANT domain is functionally distinct from the canonical Myb DNA-binding domain. Furthermore, using the Expasy tool, the hydrophobicity of the gene was analyzed. It can be seen from Figure 3C that most peaks are less than 0, which confirms that the amino acid structure tends to be hydrophilic. To further study the tertiary structure of *Dark_Pur*, phyre2 was used. With a confidence level of 100%, the *Dark_Pur* gene is similar to template ‘c6kksA’ (with 58% identity), which has six helices and the structural insights into target DNA recognition by the r2r3-type myb2 transcription factor. It is a transcription regulator activity that modulates transcription of gene sets via selective and non-covalent binding to a specific double-stranded genomic DNA sequence within a *cis*-regulatory region (Figure 3D).

Thus, the inserted gene, which we called *Dark_Pur,* was significant for the formation of purple in the materials. In order to study whether the promoter influenced gene expression, the 2000 bp promoter of the four materials was cloned, and it was observed whether there were any differences between ‘2217-Pur’ and ‘B90830’. As indicated in Figure S1, there were no different sequences between the two materials, and the sequences were also the same as the promoter of the gene in the reference genome of *B. juncea*.

### 3.3. Dark_Pur Gene Transformation in B. rapa for Functional Identification

**Figure 4:**
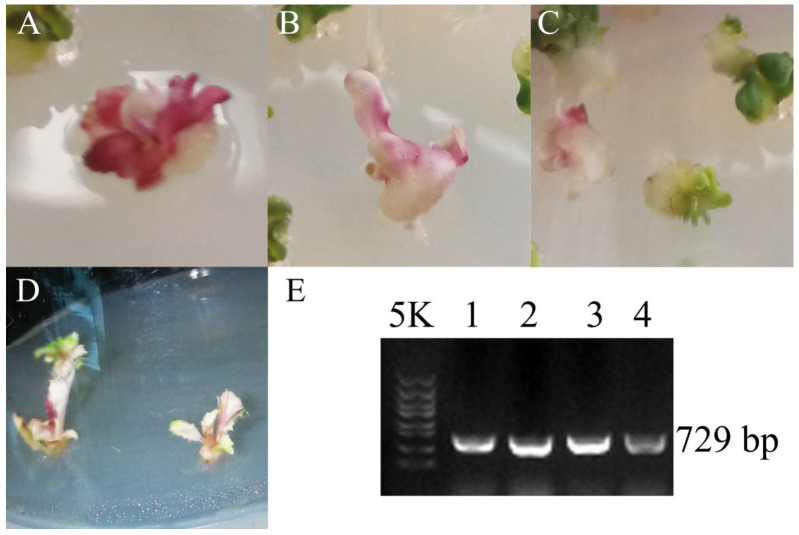
*Dark_Pur* gene transformation in *B. rapa*. (**A**–**D**): The different periods of the T0 transgenic *B. rapa*. (**E**) 1–4 represent the PCR product in T0 transgenic *B. rapa,* and the product length is 729 bp.

In order to identify whether the *Dark_Pur* gene is significant for purple color formation, a plasmid expression vector with the *Dark_Pur* gene was constructed. Using the Chinese cabbage microspores as the explant, particle bombardment transformation was applied to achieve *Dark_Pur* gene expression. About 15 d after bombardment, an embryo formed from the microspores. PCR verification and phenotype observation of the regenerated plants were performed to select transgenic plants. The primer sequences are shown in Table S1. Through culturing, purple callus and seedlings with purple leaves were observed (Figure 4). *B. rapa* turned purple in the callus period, confirming that the *Dark_Pur* gene was highly expressed. Throughout the development of T0 *B. rapa*, the leaves remained purple.

### 3.4. Transcriptomic Analysis between Purple/Green B. rapa and B. juncea

#### 3.4.1. Quality Control of the Transcriptomic Data

In order to determine whether the *Dark_Pur* induced the purple coloration mechanism of *B. rapa*, transcriptomic analysis was performed. The three periods used for the phenotyping of each group included the cotyledon stage, seedling stage, and large-leaf stage.

The transcriptome analysis of the 18 samples from three periods of *B. rapa* resulted in a total of 130.56 Gb clean data, while a total of 128.23 Gb clean data were obtained in 18 samples of *B. juncea*. There were 6.18 Gb and 5.93 Gb clean data of each sample in *B. rapa* and *B. juncea*, and the Q30 base percentage was 93.20% and 92.94% and above in *B. rapa* and *B. juncea,* respectively. The clean reads comparison efficiency ranged from 82.88% to 91.45% and 78.16% to 92.30% in *B. rapa* and *B. juncea,* respectively (Table S3). New gene discovery, alternative splicing prediction analysis, and gene structure optimization analysis were performed using the results of the comparison. A total of 3814 new genes were discovered, and 2347 were functionally annotated in *B. rapa,* while in *B. juncea*, 18,390 new genes were discovered, and 13,973 were functionally annotated. Based on the comparison results, gene expression level analysis was performed. Differentially expressed genes (DEGs) were identified and subjected to functional annotation and enrichment analysis.

The uniquely mapped reads for each sample (Table S4) of *B. rapa* and *B. juncea* were processed using HISAT2 and String Tie to identify the numbers of transcripts as the fragments per kilobase of transcript per million mapped reads (FPKM) value. According to the principal component analysis (PCA) and Pearson’s correlation analysis (Figure S2), the biological replicates of *B. rapa* and *B. juncea* were adequate. The RNA-seq data quality and the sequencing depth were able to meet the further analysis, and the variation between the different groups was sufficient to identify DEGs.

#### 3.4.2. DEG Analysis between *B. rapa* and *B. juncea*

**Figure 5:**
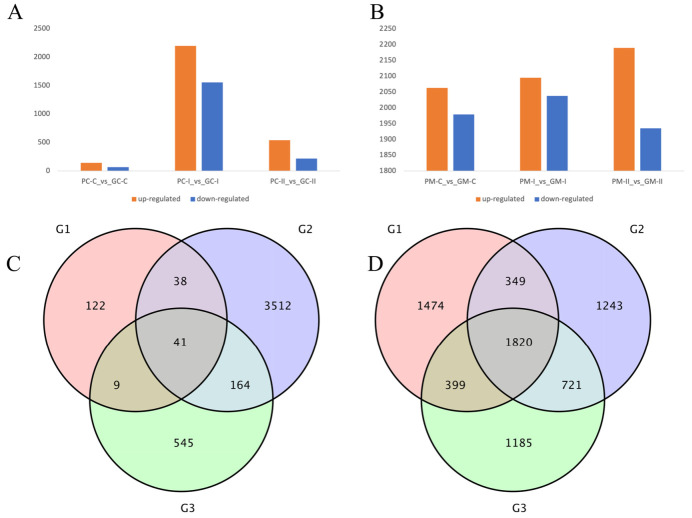
Gene expression profile of different *B*. *rapa* and *B. juncea* genotypes during different periods of B. *rapa* (**A**) and *B. juncea* (**B**). (**A**,**B**) Number of up- and down-regulated DEGs in each comparison. PC represents the purple *B. rapa* ‘2217-Pur’, GC represents the green *B. rapa* ‘2217-Gre’, PM represents the purple *B. juncea* ‘B90830’, GM represents the green *B. juncea* ‘2116’, C represents the cotyledon stage, I represents the seedling stage, and II represents the large-leaf stage. (**C**,**D**) Venn diagrams of DEGs during the three periods of B. *rapa* (**C**) and *B. juncea* (**D**). G1, G2, and G3 in (**C**) represent PC-C_vs_GC-C, PC-I_vs_GC-I, and PC-II_vs_GC-II, and G1, G2, and G3 in (**D**) represent PM-C_vs_GM-C, PM-I_vs_GM-I, and PM-II_vs_GM-II, respectively. Fold-change ≥ 2 and FDR < 0.01 were used as the criteria.

Differential expression analysis of two groups of *B. rapa* and two groups of *B. juncea* was performed using DESeq2 [40]. A fold-change of ≥2 and false discovery rate (FDR) < 0.01 were used as the screening criteria for differential expression. After analyzing the statistics of *B. rapa* in the three periods, the purple group and the green group were compared. For *B. rapa* ‘2217-Pur’ and ‘2217-Gre’, a total of 210 genes (142 up-regulated and 68 down-regulated), 3755 genes (2199 up-regulated and 1556 down-regulated), and 759 genes (540 up-regulated 219 down-regulated) in PC-C_vs_GC-C, PC-I_vs_GC-I, and PC-II_vs_GC-II were detected, respectively (Figure 5A). The results showed that the DEGs rapidly increased in period II. The DEGs in the *B. juncea* materials ‘B90830’ and ‘2116’ were also detected. There were 4042 genes (2063 up-regulated and 1979 down-regulated), 4133 genes (2095 up-regulated and 2038 down-regulated), and 4125 genes (2190 up-regulated and 1935 down-regulated) in PM-C_vs_GM-C, PM-I_vs_GM-I, and PM-II_vs_GM-II, respectively (Figure 5B). In contrast to *B. rapa*, the DEGs changed slightly in the three periods. From the Venn diagram, there are 41 genes that were always expressed in the three periods of *B. rapa* (Figure 5C), and 3512 genes belonged to PC_I. There were also 1820 genes expressed in the three periods of *B. juncea*, and the unique DEGs in the three periods were almost the same, with 1474, 1243, and 1185 genes being unique to PM-C_vs_GM-C, PM-I_vs_GM-I, and PM-II_vs_GM-II, respectively (Figure 5D).

#### 3.4.3. Transcription Factors Analysis of Anthocyanin Synthesis

**Figure 6:**
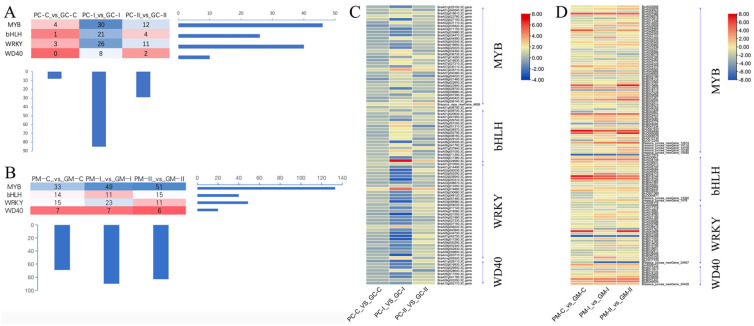
Different transcription factors in *B. rapa* and *B. juncea*. (**A**, **B**) The numbers of transcription factors (MYB, bHLH, WRKY, WD40) in (**A**) *B. rapa* (‘2217-Pur’_vs_’2217-Gre’) and (**B**) *B. juncea* (‘B90830’_vs_’2116’). (**C**) Heatmap showing the expression fold-change (Log2 fold-change) between ‘2217-Pur’ and ‘2217-Gre’ for the genes encoding transcription factors. (**D**) Heatmap showing the expression fold-change (Log2 fold-change) between ‘B90830’ and ‘2116’ for the genes encoding transcription factors.

Transcription factors (TF) are the major regulators of gene expression profiles. Therefore, focusing on the major TF families that were differentially expressed between ‘2217-Pur’ and ‘2217-Gre’ (Tables S5 and S6), recent studies have revealed that the regulatory complex is not just a simple MBW complex, but a WMBW complex in which WRKY factors together with the MBW complex regulate the anthocyanin biosynthetic pathway [11]. Our analysis showed that four TF families modulated the global gene expression levels among the two *B. rapa* groups (Figure 6A,C), showing that TFs are the main drivers of gene expression changes, leading to differential leaf coloration in *B. rapa*. The MYB, bHLH, WRKY, and WD40 families showed active members involved in gene regulation (Figure 6A,C), and therefore these TF families may be crucial for the regulation of structural genes involved in *B. rapa* skin coloration. The gene *BraA09g028560.3C* (bHLH-MYC and R2R3-MYB TF N-terminal) was strongly up-regulated over most of the developmental stages in *B. rapa* ‘2217-Pur’. The blast against *A. thaliana AT4G09820*, which is a *TT8* regulation factor that acts in a concerted manner with *TT1, PAP1,* and *TTG1* to regulate flavonoid pathways (specifically proanthocyanin and anthocyanin biosynthesis), revealed an identify of 74.296%. Additionally, *BraA03g019460.3C* (WRKY, mutants are defective in proanthocyanin synthesis and seed mucilate deposition) and *BraA07g035710.3C* (MYB-like) were up-regulated in *B. rapa* ‘2217-Pur’. Genes such as *BraA04g021550.3C*, *BraA02g040310.3C*, and *BraAnng003710.3C*, which confer resistance, were also differentially expressed, suggesting that the accumulation of anthocyanins is related to increased resistance.

Regarding *B. juncea*, the major TF families that were differently expressed between ‘B90830’ and ‘2116’ (Tables S5 and S6) were also studied. There were some MYB, bHLH, WRKY, and WD40 TFs in the *B. juncea* materials (Figure 6B,D). The genes *BjuO006089* (MYB) and *BjuB010898* (MYB) were strongly up-regulated over most of the developmental stages in *B. juncea* ‘B90830’. These genes shared 57.371% identity with *AT1G66370* (ATMYB 113), which plays a role in encoding a member of the MYB family of TFs and is involved in regulating anthocyanin biosynthesis, affecting the expression of enzymes involved in later steps of anthocyanin biosynthesis in *Arabidopsis*. The gene *BjuO006089* is located at Contig3878 9762 10938 +, and *BjuB010898* is located at B05 53425172 53426356 +, and the two genes have the same sequences.

Comparing the three genes of *B. rapa* and the two genes of *B. juncea* with the *Dark_Pur* gene, there was low similarity among the six sequences, except for the two sequences of *B. juncea,* which were the same. Regarding the three *B. rapa* genes, the highest identify of 44.21% was observed between *BraA09g028560.3C* and *BraA03g019460.3C*. Comparing *B. juncea* with the three *B. rapa* genes, the identities of *BraA07g035710.3C* and *BjuO006089* (*BjuB010898*) was 47.86%. The two sequences of *B. juncea* were the same as the former gene *Dark_Pur* (Figures S3 and S4; Table S7). Previous studies showed that *BjuB010898* is a key gene of purple *B. juncea* leaf formation [33].

#### 3.4.4. Gene Functional Annotation and Enrichment Analysis by GO and KEGG

Functional annotation of the DEGs of *B. rapa* was performed. There were totals of 202, 3713, and 746 genes annotated in the PC-C_vs_GC-C, PC-I_vs_GC-I, and PC-II_vs_GC-II groups, respectively (Table S8). For *B. juncea,* there were 3595, 3704, and 3719 genes in the PM-C_vs_GM-C, PM-I_vs_GM-I, and PM-II_vs_GM-II groups, respectively.

The gene ontology (GO) annotation system contains three main branches: biological process, cellular component, and molecular function. The statistical results of the GO classification of the DEGs are shown in Figure S5. The top three processes of the biological process, cellular component, and molecular function are shown. In the PC-C_vs_GC-C period of *B. rapa*, the DEGs in biological process belonged to the cellular process, metabolic process, and single-organism process; the DEGs in cellular component belonged to the cell, cell part, and organelle; and the DEGs in molecular function belonged to the binding, catalytic, and transporter activity (Figure S5A). In the PC-I_vs_GC-I period of *B. rapa*, the DEGs in cellular component belonged to the cell, cell part, and membrane, while the DEGs in the biological process and molecular function were the same as for the first period (Figure S5B). In the PC-II_vs_GC-II period of *B. rapa*, the DEGs in biological process belonged to the cellular process, metabolic process, and single-organism process; the DEGs in the cellular component belonged to the cell, cell part, and membrane; and the DEGs in the molecular function belonged to the binding, catalytic, and nucleic acid binding TF activity (Figure S5C). The DEGs in the three periods were basically unchanged. Regarding *B. juncea*, the DEGs in the biological process belonged to the cellular process, metabolic process, and single-organism process; the DEGs in the cellular component belonged to the cell, cell part, and membrane; and the DEGs in the molecular function belonged to the binding, catalytic, and transporter activity (Figure S5D) in the first period. The second and the third periods of *B. juncea* were the same as the first period (Figure S5E,F).

**Figure 7:**
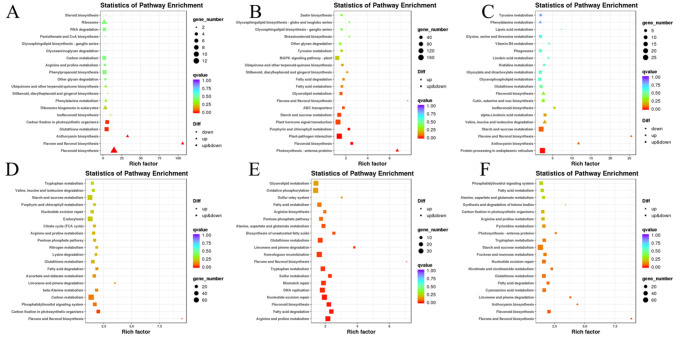
Enrichment analysis of DEGs in KEGG pathways. (**A**–**C**) DEGs of *B. rapa* in PC-C_vs_GC-C, PC-I_vs_GC-I, PC-II_vs_GC-II groups. (**D**–**F**) DEGs of *B. juncea* in PM-C_vs_GM-C, PM-I_vs_GM-I, PM-II_vs_GM-II groups.

As leaf color is linked to anthocyanin accumulation, the flavonoid and anthocyanin biosynthesis Kyoto Encyclopedia of Genes and Genomes (KEGG) pathways in the three periods (Table S9) were focused on. The anthocyanin biosynthesis (ko00942), flavonoid biosynthesis (ko00941), isoflavonoid biosynthesis (ko00943), and flavone and flavonol biosynthesis (ko00944) pathways were selected, and the genes involved in these pathways are indicated in Figure 7. There were three genes that belonged to anthocyanin biosynthesis (ko00942) in *B. rapa*, namely, *BraA06g021610.3C* (UGT), *BraA08g009740.3C* (UGT), and *BraA10g012180.3C* (UGT). These three genes are structural genes and late biosynthetic genes that are essential for producing anthocyanins and some specific flavonoids. Using the same analysis method, six anthocyanin biosynthesis (ko00942) genes in *B. juncea* were found, namely, *BjuA028394, BjuO011591, Brassica_juncea_newGene_3722, Brassica_juncea_newGene_3723, Brassica_juncea_newGene_8857,* and *Brassica_juncea_newGene_8858* (Table S10).

### 3.5. The DEGs from the Transcriptome Analysis Were Detected by qRT-PCR

**Figure 8:**
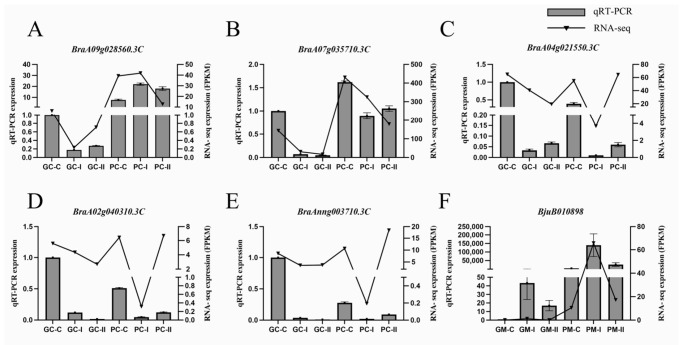
Relative expression of 6 selected DEGs analyzed by qRT-PCR and RNA-seq expression trends. (**A**–**F**) The expression level of *BraA09g028560.3C*, *BraA07g035710.3C*, *BraA04g021550.3C, BraA02g040310.3C*, and *BraAnng003710.3C* and *BjuB010898*. Bar and line graphs represent the qRT-PCR and RNA-Seq data, respectively. Data are presented as the mean ± standard error (SE).

To verify the results of the RNA-Seq analysis, five DEGs were verified using qRT-PCR in *B. rapa*, including two TFs, which related to the anthocyanin synthesis pathway (*BraA09g028560.3C*, *BraA07g035710.3C*), and three TFs, which conferred resistance (*BraA04g021550.3C, BraA02g040310.3C*, and *BraAnng003710.3C*) (Figure 8A–E). The gene *BjuB010898*, which related to anthocyanin synthesis, was verified using qRT-PCR in *B. juncea* (Figure 8F). The RNA-seq analysis and the results from qRT-PCR were consistent during three periods in *B. rapa* and *B. juncea,* indicating the reliability of transcriptome sequencing. The expression levels of the genes that confer anthocyanin synthesis pathway were higher in purple material than in green material, which were the same as the RNA-seq data, demonstrating that these genes are responsible for the purple color formation.

### 3.6. Metabolite Profiling Analysis between Purple/Green B. rapa and B. juncea

**Figure 9:**
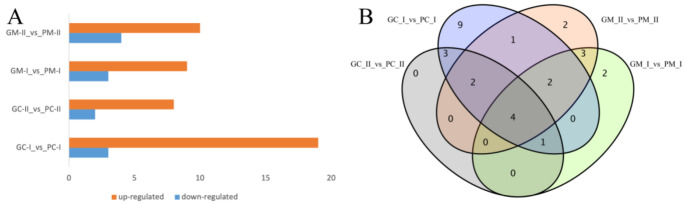
Metabolite expression profiles of different *B. rapa* and *B. juncea* genotypes during different periods. (**A**) Numbers of up- and down-regulated DEMs in each comparison. PC represents the purple *B. rapa* ‘2217-Pur’ and GC represents the green *B. rapa* ‘2217-Gre’, PM represents the purple *B. juncea* ‘B90830’ and GM represents the green *B. juncea* ‘2116’. (**B**) Venn diagrams of differentially expressed metabolites (DEMs) during the two periods in *B. rapa* and *B. juncea*.

The color of *B. rapa* and *B. juncea* is produced by plant pigments, and anthocyanins are typically responsible for purple coloration. Using the LC-MS/MS platform, qualitative and quantitative metabolome analyses were performed on 12 *B. rapa* samples (PC-I, PC-II, GC-I, GC-II) and 12 *B. juncea* samples (PM-I, PM-II, GM-I, GM-II), and a total of 108 metabolites was detected. First, the DEMs in *B. rapa* were investigated and detected; there were 22 (19 up-regulated and 3 down-regulated) and 10 (8 up-regulated and 2 down-regulated) DEMs in PC-I_vs_GC-I and PC-II_vs_GC-II, respectively. In *B. juncea*, there were 12 (9 up-regulated and 3 down-regulated) DEMs and 14 (10 up-regulated and 4 down-regulated) DEMs in PM-I_vs_GM-I and PM-II_vs_GM-II, respectively (Figure 9A). Using a Venn diagram, the DEMs in PC-II_vs_GC-II of *B. rapa* were the same as PC-I_vs_GC-I in *B. rapa*. Most DEMs were the same in the two comparison groups, and there were three and five unique DEMs in PM-I_vs_GM-I and PM-II_vs_GM-II, respectively (Figure 9B). In order to determine why *B. rapa* and *B. juncea* appeared in different shades of purple at the same time, the DEMs in PC-I_vs_GC-I and PM-I_vs_GM-I were analyzed. There were 7 DEMs that were the same in the two groups, 15 DEMs were unique to PC-I_vs_GC-I, and 5 DEMs were unique to PM-I_vs_GM-I. Analyzing the DEMs between PC-II_vs_GC-II and PM-II_vs_GM-II, six DEMs were the same, and there were four and eight unique DEMs in PC-II_vs_GC-II and PM-II_vs_GM-II, respectively. The unique DEMs may be the key metabolites responsible for the different coloration of the two materials.

**Figure 10:**
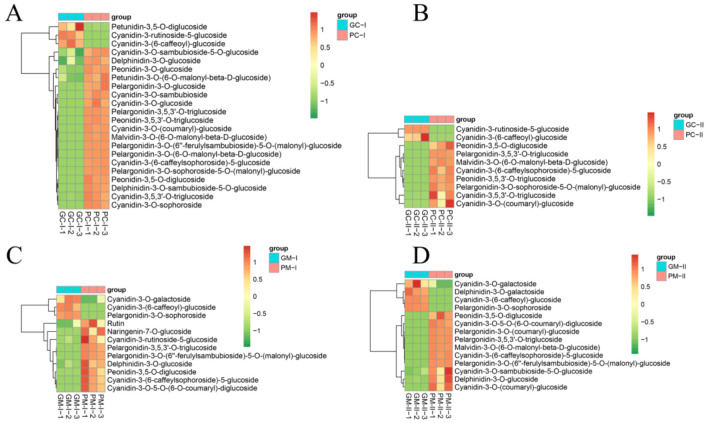
DEMs in *B. rapa* leaf skins during development. (**A**,**B**) Heatmaps of DEMs in the PC-I_vs_GC-I and PC-II_vs_GC-II comparisons. (**C**,**D**) Heatmaps of DEMs in the PM-I_vs_GM-I and PM-II_vs_GM-II comparisons.

Compared with the green materials, the differential metabolites in purple *B. rapa* and in purple *B. juncea* were cyanidins and pelargonidins. There were nine, five, five, and six differential cyanidins in PC-I_vs_GC-I, PC-II_vs_GC-II, PM-I_vs_GM-I, and PM-II_vs_GM-II, respectively. There were five, two, three, and four different pelargonidins in PC-I_vs_GC-I, PC-II_vs_GC-II, PM-I_vs_GM-I, and PM-II_vs_GM-II, respectively (Table S11). The DEMs of *B. rapa* in PC-I_vs_GC-I indicated that cyanidin-3,5,3’-O-triglucoside, delphinidin-3-O-glucoside, and cyanidin-3-O-(coumaryl)-glucoside were more abundant than the others. The DEMs of *B. rapa* in PC-II_vs_GC-II indicated that cyanidin-3-O-(coumaryl)-glucoside and cyanidin-3,5,3’-O-triglucoside were more abundant than the others. Delphinidin-3-O-glucoside, rutin, and cyanidin-3-O-5-O-(6-O-coumaryl)-diglucoside were higher in PM-I_vs_GM-I, and delphinidin-3-O-glucoside and cyanidin-3-O-(coumaryl)-glucoside were higher in PM-II_vs_GM-II (Figure 10). Studying the different DEMs in the PC-I_vs_GC-I and PM-I_vs_GM-I group, the major unique metabolites were cyanidins (cyanidin-3,5,3’-O-triglucoside, cyanidin-3-O-(coumaryl)-glucoside, cyanidin-3-O-glucoside, cyanidin-3-O-sambubioside, cyanidin-3-O-sambubioside-5-O-glucoside, cyanidin-3-O-sophoroside) in PC-I_vs_GC-I, and the unique metabolites were naringenin-7-O-glucoside, rutin, cyanidin-3-O-galactoside, cyanidin-3-O-5-O-(6-O-coumaryl)-diglucoside, and pelargonidin-3-O-sophoroside in PM-I_vs_GM-I. As for PC-II_vs_GC-II and PM-II_vs_GM-II, the unique metabolites included cyanidin-3-rutinoside-5-glucoside, cyanidin-3,5,3’-O-triglucoside, peonidin-3,5,3’-O-triglucoside, pelargonidin-3-O-sophoroside-5-O-(malonyl)-glucoside in the PC-II_vs_GC-II group and cyanidin-3-O-5-O-(6-O-coumaryl)-diglucoside, cyanidin-3-O-galactoside, cyanidin-3-O-sambubioside-5-O-glucoside, delphinidin-3-O-galactoside, delphinidin-3-O-glucoside, pelargonidin-3-O-(6’’-ferulylsambubioside)-5-O-(malonyl)-glucoside, pelargonidin-3-O-(coumaryl)-glucoside, and pelargonidin-3-O-sophoroside in the PM-II_vs_GM-II group (Table S12). Over time, purple Chinese cabbage attains a more obvious purple phenotype (Figure 1). The unique differential metabolites in stage II of *B. rapa* and *B. juncea* were analyzed. There were fewer unique DEMs in PC-II_vs_GC-II than in PM-II_vs_GM-II. Comparing the unique DEMs in PC-II_vs_GC-II with PC-I_vs_GC-I, three metabolites were found, namely, cyanidin-3,5,3’-O-triglucoside, peonidin-3,5,3’-O-triglucoside, and pelargonidin-3-O-sophoroside-5-O-(malonyl)-glucoside. These may be the major metabolites responsible for the darker purple color of *B. rapa* than *B. juncea*. In the PC-I_vs_GC-I period of *B. rapa*, there was one compound, peonidin-3-O-glucoside, which was annotated to the anthocyanin biosynthesis (ko00942) pathway. Regarding PM-I_vs_GM-I of *B. juncea*, rutin was annotated to the biosynthesis of secondary metabolites (ko01110), flavone and flavonol biosynthesis (ko00944), and metabolic pathways (ko01100) (Table S13).

## 4. Discussion

### 4.1. The Structure Analysis and Transformation of the Dark_Pur Gene

As a water-soluble pigment, anthocyanins determine the formation of plant color. They have also received extensive attention from researchers due to their health properties [46,47]. In plants, anthocyanins are crucial for pink, red, and purple color formation. Anthocyanins are distributed across tissues, such as the leaves, petals, curds, leaf-head, and rhizomes [31,48,49,50,51,52,53]. The mechanism of purple color formation in *Brassica* was explored herein. Some candidate genes for purple color formation in *Brassica* were found [24,54,55]. The location of the genes that regulate the purple color of *Brassica* leaves was also determined, including genes located at A02, A03, A07, and A09 [24,25,26,34]. We previously found a transcript named *c3563g1i2*, and based on this former study, named it *Dark_Pur* and cloned the *Dark_Pur* gene, which may be responsible for the purple coloration of Chinese cabbage [30]. Sequence analysis showed that the *Dark_Pur* gDNA sequence of ‘2217-Pur’ and ‘B90830’ was the same, and the sequence was the same as in the reference genome of *B. juncea*, which indicated that the *Dark_Pur* gene originated from mustard through hybridization. Though the green mustard ‘2116’ had the 1188 bp length gDNA segment of *Dark_Pur*, the cDNA of the *Dark_Pur* in mustard ‘2116’ that showed the green appearance could not be obtained. It is possible that the gDNA was not transcribed into cDNA and was not translated into a functional protein, and thus did not form a purple phenotype. The purple traits of Chinese cabbage are mainly derived from purple-red mustard, violet pak choi, purple cai-tai, and purple-red turnip. Our materials originated from purple mustard. Further study of the structure of the *Dark_Pur* gene revealed two SANT regions, which is similar to *BrMYB2* [31]. The protein tertiary structure analysis indicated that the *Dark_Pur* gene was similar to ‘c6kksA’, which has six helices and the structural insights into target DNA recognition by the r2r3-type myb2 TF. This further confirmed that the *Dark_Pur* gene plays an important role in the purple formation of cabbage leaves. Research on the 2000 bp promoter before *Dark_Pur* did not demonstrate any differences between *B. rapa* and *B. juncea*. The sequence before the 2000 bp promoter should be analyzed to determine if there are important regions for purple color formation. To further study whether the candidate gene is responsible for the purple color of *B. rapa*, a plasmid was designed for transformation. Through particle bombardment and microspore culture, purple *B. rapa* was obtained. This indicated that the *Dark_Pur* gene was responsible for the purple color. Using the microspores as the receiver, the transgenic offspring were all in the DH (double haploid) group. With DH lines, continuous selfing is not necessary to obtain homozygous plants if positive transgenic offspring are found; this would have greatly reduced the experiment time.

### 4.2. Transcriptomic Analysis of B. rapa and B. juncea

During growth, *B. rapa* showed darker purple coloration than *B. juncea*. Purple Chinese cabbage and green Chinese cabbage were obtained from the 2217 inbred line. The background was relatively homozygous. Due to the lower background interference, the DEGs may have a high correlation with the anthocyanin synthesis in *B.rapa*. During the PC-I_vs_GC-I period, the number of DEGs in cabbage increased significantly. The genetic background of purple mustard and green mustard is quite different, and so the DEGs in the different periods were more numerous than in Chinese cabbage, and the number of DEGs in each period exceeded 4000. When the accumulation of anthocyanins increases, chlorophyll synthesis will be impacted, and so some of the down-regulated genes may be related to chlorophyll synthesis [56].

R2R3-MYB, bHLH, and WD40 (MBW complex) usually control the structural genes of anthocyanin formation, and the MYB TFs are typically crucial in anthocyanin biosynthesis [5,57,58,59]. In this study, the MBW TFs in *B. rapa* and *B. juncea* were analyzed and it was found that *BraA09g028560.3C* (bHLH-MYC and R2R3-MYB TF N-terminal) was strongly up-regulated over most of the developmental stages in *B. rapa* ‘2217-Pur’. This gene is similar to *A. thaliana AT4G09820,* which is a TT8 regulation factor that plays a role in flavonoid pathways. A previous study showed that, with the exception of the MBW complex, WRKY also plays an important role in the anthocyanin pathway [60]. In this study, *BraA03g019460.3C* (WRKY) and *BraA07g035710.3C* (MYB-like) were also up-regulated in *B. rapa* ‘2217-Pur’. Genes such as *BraA04g021550.3C*, *BraA02g040310.3C,* and *BraAnng003710.3C,* which confer resistance, were also differentially expressed, suggesting that the accumulation of anthocyanins is related to increased resistance [61]. In *B. juncea*, the genes *BjuB010898* (MYB) and *BjuO006089* (MYB) were strongly up-regulated over most of the developmental stages in *B. juncea* ‘B90830’. These genes are similar to *AT1G66370* (ATMYB 113), which plays a role in encoding a member of the MYB family of TFs and is involved in regulating anthocyanin biosynthesis in *Arabidopsis*. The sequence alignment results showed that the *Dark_Pur* gene sequence of purple Chinese cabbage was the same as the mustard purple gene *BjuB010898* (MYB). During the mustard–Chinese cabbage hybridization process, the insertion of foreign *Dark_Pur* gene from purple mustard significantly may increase the expression of *BraA09g028560.3C, BraA03g019460.3C* and *BraA07g035710.3C* genes in purple Chinese cabbage, resulting in a purple phenotype in purple Chinese cabbage. However, any clear evidence of how the *Dark_Pur* genes interact with other TFs or structure genes was not found. A future study should focus on elucidating the *Dark_Pur* gene transport mechanism from mustard to Chinese cabbage.

### 4.3. The Expression Level of the Structure Genes of the B. rapa

**Figure 11:**
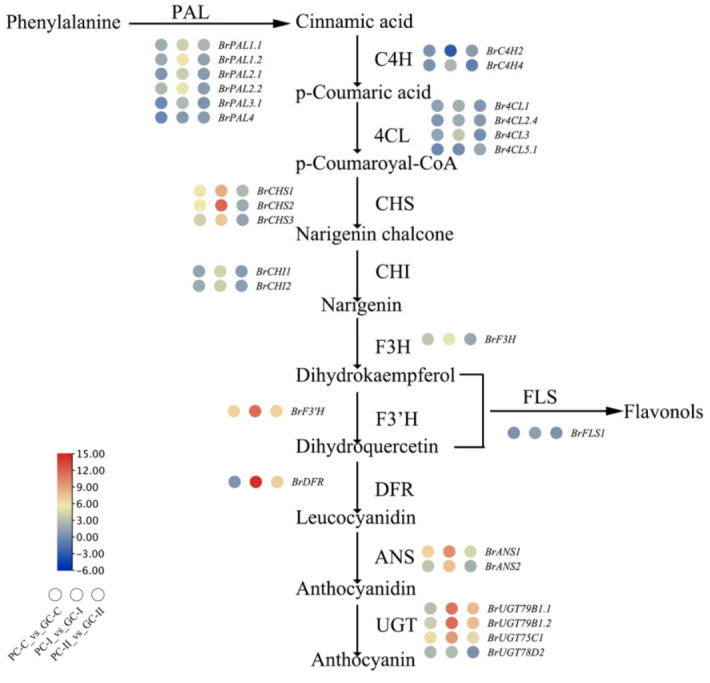
Transcriptional regulatory network. Anthocyanin transcriptional regulatory network in *B. rapa*.

Structural genes play an important role in the anthocyanin biosynthesis pathway. From previous reports on purple plants, structural anthocyanin biosynthesis genes also combine with TFs, and a single MYB TF can activate the expression of EBGs, whereas the LBGs are regulated by a triple complex consisting of the MYB, bHLH, and WD40 TFs [62,63,64,65,66]. Generally, anthocyanins (cyanidin, pelargonidin, and delphinidin derivatives) are synthesized via three main branches of the biosynthetic pathway [16]. Comparative genomics with collinearity and non-collinearity homologous gene analysis was used to identify homologous anthocyanin synthesis genes in Chinese cabbage based on the Chinese cabbage genome (v1.5) [14]. The expression levels of structural genes (Table S14) were compared in our experiment, and a heatmap of the expression levels of metabolic pathway genes was illustrated (Figure 11). With the exception of *BrPAL3.1, BrPAL4,* and *Br4CL5.1* in the GC-C_vs_PC-C period, *BrC4H2* in the PC-I_vs_GC-I period, and *BrC4H4* in the PC-II_vs_GC-II period, the *PAL, C4H, 4CL, CHS, CHI, F3H, F3’H, FLS, DFR, ANS,* and *UGT* genes were up-regulated in the three periods. The *CHS, CHI, F3H, F3’H,* and *FLS* genes are EBGs that are usually regulated by R2R3-MYB TFs, while *DFR, ANS,* and *UGT* are usually regulated by the MBW (MYB-bHLH-WD40 ternary complex) [14,15].

### 4.4. KEGG Annotation of the B. rapa and B. juncea

The KEGG database provides integrated metabolic pathway queries, including the metabolism of carbohydrates, nucleosides, and amino acids, and the biodegradation of organic matter. Through GO and KEGG analysis, the anthocyanin biosynthesis (ko00942), flavonoid biosynthesis (ko00941), isoflavonoid biosynthesis (ko00943), and flavone and flavonol biosynthesis (ko00944) pathways were annotated in the two species. The genes *BraA06g021610.3C* (UGT), *BraA08g009740.3C* (UGT), and *BraA10g012180.3C* (UGT) were annotated in the anthocyanin biosynthesis (ko00942) pathway in *B. rapa*, and *BjuA028394*, *BjuO011591*, *Brassica_juncea_newGene_3722, Brassica_juncea_newGene_3723, Brassica_juncea_newGene_8857,* and *Brassica_juncea_newGene_8858* were annotated in *B. juncea*.

### 4.5. Metabolome Analysis of the B. rapa and the B. juncea

Using LC-MS/MS, different metabolites were found in the two species. The differential metabolites included cyanidins and pelargonidins in purple *B. rapa* and *B. juncea*, which differs from findings in a previous study [30]. To further determine why Chinese cabbage and mustard exhibited different phenotypes, the DEMs in PC-I_vs_GC-I and PM-I_vs_GM-I were compared. The DEMs in PC-II_vs_GC-II and PM-II_vs_GM-II were also compared. The unique DEMs may be the key metabolites and responsible for the different colors of the two materials.

In summary, this research analyzed the structure of the *Dark_Pur* gene and, using the bombardment method, verified the function of the *Dark_Pur* gene. Through transcriptome analysis, we confirmed that the *Dark_Pur* gene can improve the expression level of the TFs, such as *BraA09g028560.3C*, *BraA03g019460.3C* and *BraA07g035710.3C,* and is responsible for the purple color in *B. rapa*. Metabolome analysis showed the different metabolites between purple and green *B.rapa* and *B.juncea* and provided evidence that the unique DEMs may be responsible for the purple *B.rapa* and *B.juncea* coloration. This study provides additional information for further studies of anthocyanins in *B. rapa*.

## Data Availability

The data were submitted to the NCBI database, and the accession number is PRJNA770000 (https://www.ncbi.nlm.nih.gov/search/all/?term=PRJNA770000, accessed on 10 November 2021).

## Supplementary Materials

Figure S1. Promoter sequence comparison between ‘2217-Pur’ and ‘B90830’; Figure S2. Good biological replication of *B. rapa* and *B. juncea.* A. Principal component analysis (PCA) of the *B. rapa* RNA-seq data. B. PCA of the *B. juncea* RNA-seq data. C. Cluster heatmap of different samples of *B. rapa*. D. Cluster heatmap of different samples of *B. juncea.*; Figure S3. Multiple sequence alignment among genes. *Dark_Pur, BraA09g028560.3C, BraA03g019460.3C,* and *BraA07g035710.3C* are candidate genes of *B. rapa. BjuB010898* (*BjuO006089*) are candidate genes of *B. juncea.*; Figure S4. Phylogenetic tree of the six genes.; Figure S5. GO annotation of the materials. A, B, C. GO annotation of *B. rapa* in PC-C_vs_GC-C, PC-I_vs_GC-I, PC-II_vs_GC-II groups. D, E, F. GO annotation of *B. juncea* in PM-C_vs_GM-C, PM-I_vs_GM-I, PM-II_vs_GM-II groups. Table S1. The primer of *Dark_Pur*. Table S2. Specific primers for differential gene sequences for qRT-PCR. Table S3. Transcriptome sequencing data quality and output statistics. Table S4. Mapping information of 18 samples of the Chinese cabbage and 18 samples of the mustard. Table S5. Transcription factors in different periods of Chinese cabbage and mustard. Table S6. Filtered transcription factors and log2 FC in different periods of Chinese cabbage and mustard. Table S7. The identity of six candidate genes. Table S8. Gene functional annotation in materials. Table S9. KEGG pathway of three periods of Chinese cabbage and mustard. Table S10. The gene annotation of anthocyanin accumulation of Chinese cabbage and mustard. Table S11. Different metabolites in purple and green Chinese cabbage and mustard. Table S12. The DEMs in Chinese cabbage and mustard comparison group. Table S13. DEMs in KEGG pathway. Table S14. Gene name and ID change in different species and version.
