# Supplementary figures and images for: Comprehensive Transcriptome–Metabolome Analysis and Evaluation of the Dark_Pur Gene from Brassica juncea that Controls the Differential Regulation of Anthocyanins in Brassica rapa
Source: Genes (Basel). 2022 Jan 31;13(2):283. doi: 10.3390/genes13020283 (PMC8871995; doi:10.3390/genes13020283)

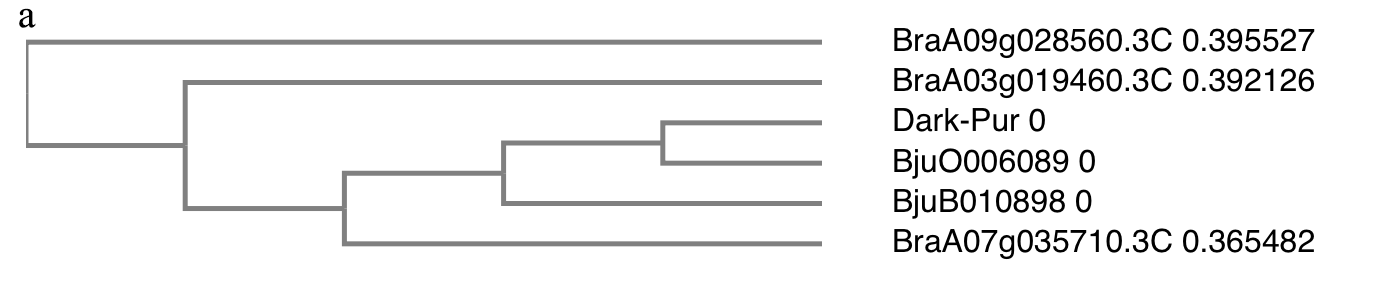

Supplement: Supplementary file 1 [file genes-13-00283-s001.zip › Fig S4.tif]

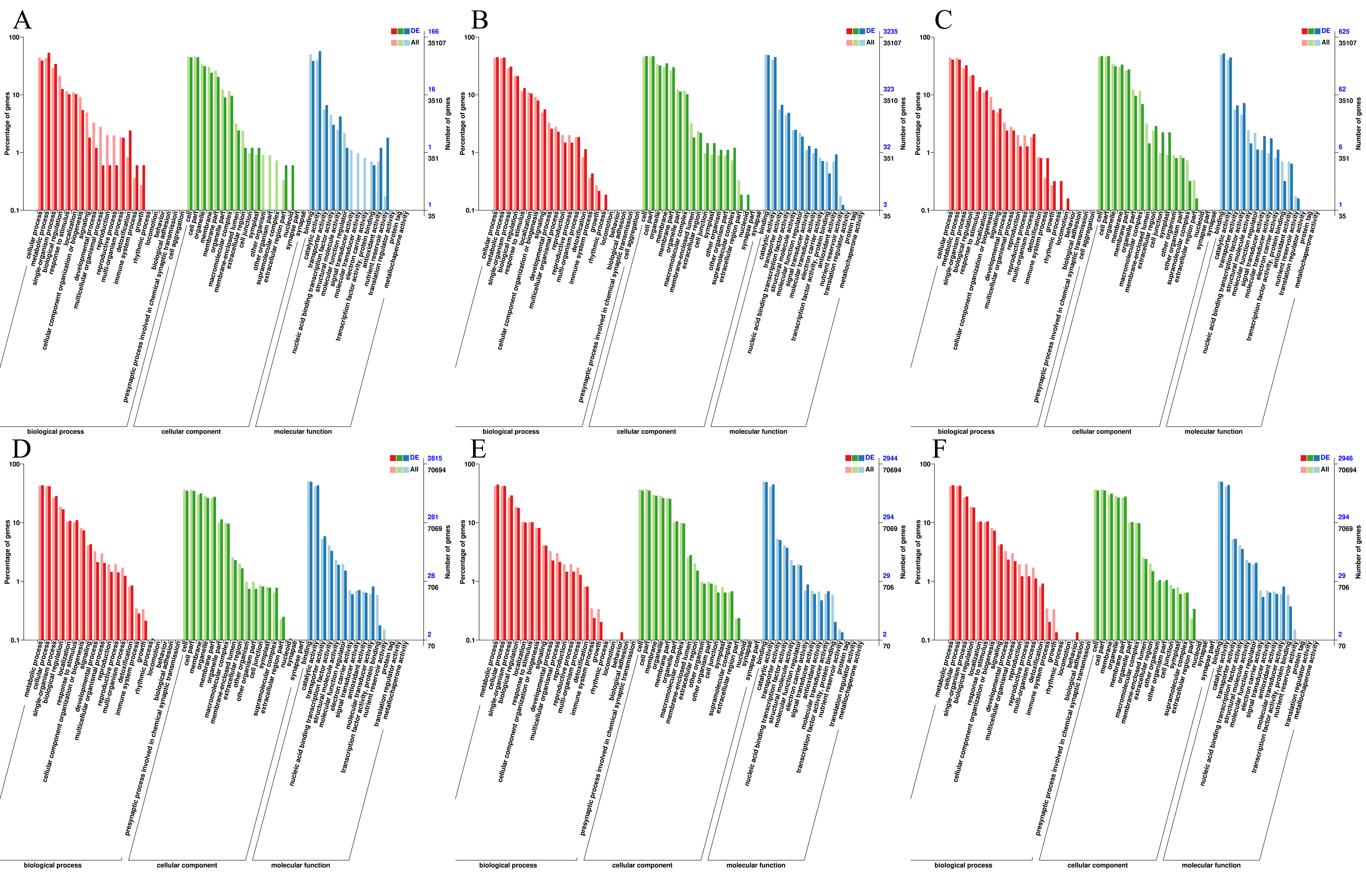

Supplement: Supplementary file 1 [file genes-13-00283-s001.zip › Fig S5.tif]
